# Minding the Gaps in Fish Welfare: The Untapped Potential of Fish Farm Workers

**DOI:** 10.1007/s10806-021-09869-w

**Published:** 2021-09-27

**Authors:** Christian Medaas, Marianne E. Lien, Kristine Gismervik, Tore S. Kristiansen, Tonje Osmundsen, Kristine Vedal Størkersen, Brit Tørud, Lars Helge Stien

**Affiliations:** 1 Department of Social Anthropology, University of Oslo, Oslo, Norway; 2 Norwegian Veterinary Institute, Oslo, Norway; 3 Research Group Animal Welfare, Institute of Marine Research, Bergen, Norway; 4 NTNU Social Research, Trondheim, Norway

**Keywords:** Aquaculture, Welfare, Salmon, Norway, Regulation, Responsibility

## Abstract

The welfare of farmed fish is often regarded with less concern than the welfare of other husbandry animals, as fish are not universally classified as sentient beings. In Norway, farmed fish and other husbandry animals are legally protected under the same laws. Additionally, the legislature has defined a number of aquaculture-specific amendments, including mandatory welfare courses for fish farmers who have a key role in securing animal welfare, also with regards to noting welfare challenges in the production process. This article uses fish welfare courses as a site from which to inquire about the common-sense understanding of fish welfare in Norwegian fish farming. The focus is specifically on fish farm employees, their experiences of welfare-related issues and contradictions in their daily work, and the struggle to act responsibly in aquaculture settings. Through participant observation at welfare courses, as well as interviews and conversations with fish farm workers, the article details how challenges are experienced ‘on the ground’, and suggests how fish farm workers’ own experiential knowledge might be mobilized to improve the general welfare of farmed fish.

## Introduction

Aquaculture is the world’s fastest-growing form of animal-based food production (FAO, 2018) and one of Norway’s most lucrative industries. It is also controversial: while proponents claim that aquaculture will supplant oil as the country’s principal revenue source and refer to the economic development for coastal communities that aquaculture has enabled, others criticize the environmental impacts of intensive inshore aquaculture and cite concerns for the welfare of the fish in the sea-cages, among other issues. Statistics suggest that the Norwegian aquaculture industry experiences more loss in production than other industrial food animal production (IFAP) industries in the country. The Norwegian government has responded to these concerns by implementing welfare legislation in aquaculture based on the premise that fish are sentient beings, capable of suffering and worthy of welfare and respect (Law & Lien, 2016: 33, Lybæk, 2016). These regulations act to control various parts of the aquaculture operation, such as transport and handling of fish, the amount and density of fish individuals in sea-cages, feeding, vaccination and medical treatment, and slaughter. As part of these regulations, mandatory and regular courses in fish welfare are required of all aquaculture employees working directly with the fish, including site leaders.^1^ This article asks how Norwegian salmon farmers perceive and pursue the industry’s animal-welfare related regulations, and how mandatory courses in fish welfare frame the understanding of fish well-being amongst fish farm workers.

Through participant observation and informal interviews with participants at several fish welfare courses and aquaculture facilities along the country’s southwestern coastline, we approach these welfare courses as an empirical entry point to exploring the discourse about and distribution of responsibility for fish welfare, in a situation of capital expansion and normative change in the Norwegian aquaculture industry. How do farm workers’ responsibilities for fish welfare translate (or not) to the ability to act responsibly? To what extent might mandatory welfare courses empower farm workers within the hierarchical order of the organization, and to what extent do farm workers feel that the standards that they are taught can—or should—be implemented in practice?

The article begins with a brief history of aquaculture in Norway and its contemporary significance. We then introduce some key concepts based on current theoretical debates relevant to animal ethics and welfare, outlining the welfare-related challenges and responses from aquaculture actors that we identified in our research. Following this, we detail our research methodology, and present our findings. We suggest that while welfare courses are potentially a positive step towards improving fish welfare, they also expose certain gaps between the *ideals* described in fish welfare regulation and the shortcomings of *day-to-day* practices in the industry. In these gaps, we see the contours of other dilemmas for fish farm workers—balancing personal and professional concern for fish welfare with the requirements of an intensive occupation as well as the delegation of responsibility to farm workers for fish welfare versus workers’ ability to respond. In the final part of the article, we discuss these findings in light of current debates on human responsibility for animal welfare in domestication and the implications of their extension to aquaculture.

## Aquaculture in Norway

As in most countries along the North Atlantic rim, fisheries have been central to the livelihoods of coastal communities in Norway, as subsistence as well as for trade. Since the 1970s, however, aquaculture has become an industry of increasing importance. From their beginning as small business activities for people living along the country’s coast (Gjedrem, 1993; Lien, 2015), aquaculture in Norway has developed into a major industry (Clark & Bostock 2017; Garlock et al., 2020), dominated today by large corporations that own farms across Norway as well as abroad, in countries such as Chile, Scotland, and Canada. In 2019, the Norwegian aquaculture industry produced more than 370 million salmon individuals for ongrowing in sea-cages (Directorate of Fisheries, 2020), making salmon by far the most populous livestock animal in the country. Hailed by politicians and media as one of Norway’s fastest-growing industries, with untapped potential for future growth (Law & Lien 2019). In 2019, Norwegian aquaculture generated over $8 billion and employed thousands of people.^2^

Traditionally, there has been a significant distinction in how fish have been viewed in comparison to land animals (Ministry of Agriculture & Food, 2002), and the notion that “fish are not animals” [*fisk er ikke dyr*] has been common in Norway in the past (The Council for Animal Ethics, 2014). The idea that fish might feel pain when they are hooked or entangled in a net has hardly been a serious concern, at least not publicly. Recently, with the growth of aquaculture, this has changed (Lien, 2015; the Council for Animal Ethics, 2014).

Salmon farming in Norway occurs in many places: in laboratories, offices, boardrooms, shops, and conference halls. It most directly occurs, however, on the fish farms that skirt the country’s extensive coastline. These locations are often remote and only accessible by boat, but are a significant source of employment for local villagers. Here, salmon farmers, known colloquially as “ranchers” [*røktere*]—but whom we refer to in this article as fish farm workers—perform the varied, challenging, and sometimes problematic work of farming salmon. With the help of an array of techno-scientific and biomedical aids—sensors, underwater cameras, feed hoppers, pipes, tubes, nets, walkways, boats, cages, conveyor belts, pumps—they breed, raise, keep, care for, and kill millions of salmon each year.

### Challenges relevant to fish welfare

Although the work can be very profitable for fish farm workers, and exponentially more so for company owners and investors (Hage, 2019), it is often fraught with challenges (Stien et al., 2020a, 2020b). These challenges include parasites (Torrisen et al. 2013), infection, and contagion within and beyond the confines of the sea-cages (Noble et al., 2018). They also include unpredictable and often inclement weather and oceanic conditions as well as polarized and critical debates related to environment and animal welfare in aquaculture (Olsen & Osmundsen, 2017; Stien et al., 2020a; Young et al., 2019).

The challenge of sea lice in particular (Overton et al., 2018) has led to several ameliorative measures on the part of aquaculture actors, with varying degrees of success and effects on fish welfare (Barret et al., 2020). One of these measures has been the introduction of so-called “cleaner fish” into sea-cages—and into the complex system of measures implemented to improve and ensure salmon welfare in aquaculture operations. These specific species of fish (predominantly Ballan wrasse, Goldsinny wrasse, and Lumpfish), as their nickname indicates, are employed to eat sea lice that feed parasitically on the salmon. Considered a safer and more environmentally friendly way to remove sea lice than other chemical treatments, and less stressful for the salmon than mechanical or thermal treatments, the introduction of cleaner fish into sea cages nevertheless comprise novel welfare challenges of which our interlocutors repeatedly made us aware. Recent studies indicate that cleaner fish have a relatively high mortality rate (Stien et al., 2020b) and a total loss rate (undocumented mortalities and escapees) of nearly 100% (Geitung et al., 2020). They die of disease or predation, either while “on the job” in salmon pens or during long-distance transport that many of them endure to reach the salmon pens on the western coast of Norway (Størkersen & Amundsen, 2019; Stien, Størkersen & Gåsnes 2020a). In order to meet increased demands and improve biosecurity as well as to respond to the overfishing of cleaner fish populations in the wild, efforts have been made to farm cleaner fish in Norway (Amundsen & Størkersen, 2019). Nearly 30 million cleaner fish were produced in Norway in 2019 (Directorate of Fisheries, 2020). Unlike farmed salmon, cleaner fish are not the output of production as such, and have therefore remained largely invisible to consumers as well as to animal welfare activists in the country until recently. While a full discussion of cleaner fish welfare in Norwegian aquaculture falls outside of the scope of this article, we will return to the challenges that arise in relation to these most recent aquaculture “domesticates” later in the article. During welfare courses and discussions with fish farm workers, interlocutors often specifically referred to the plight of cleaner fish as an animal welfare problem that arose in the course of their own work.^3^

Norway is home to some of the world’s largest wild populations of Atlantic salmon. Because Norwegian salmon farming occurs in waterways that often constitute migratory routes for wild salmon, the challenges of salmon farming involve not only the welfare and health of farmed salmon and their human constituents. They also involve the protection of *wild* salmon, whose health is threatened by the abundance of sea lice on farmed salmon, and whose genetics are threatened by farmed salmon themselves, who occasionally escape their cages to mate (Osmundsen et al., 2020). In addition, large-scale sea-based aquaculture operations release large amounts of nutrients, organic matter, and chemicals (for example antifouling agents for nets and facilities, as well as delousing chemicals) into the open sea, thus posing potential threats to other organisms in local ecosystems. The fact that it is difficult to accurately assess the impact of some these emissions in the marine environment may contribute to public skepticism.

These and other issues are frequently discussed in polarized media debates about salmon farming (Olsen & Osmundsen, 2017; Stien et al., 2020a; Young et al., 2019), exacerbating many farm workers’ own feelings of responsibility and moral pressure. Adding to this sentiment is a continuous expectation of economic growth and industrial intensification, juxtaposed with a complex and dynamic framework of legislation (Gismervik et al., 2020; Mellbye, 2018) to which fish farm workers are continually pursuant, and spend a great deal of time and energy pursuing.

### Welfare practices and regulations

Fish farm workers spend many of their working hours alongside sea cages, maintaining the technically and logistically complex systems that in turn aspire to keep salmon healthy, resilient, growing, and satisfied—or in the words of one interlocutor, “at least free from too much unnecessary suffering”. Fish farm workers themselves are an integral part of these systems. Working at sea, exposed year-round to the elements, their work is mentally and physically demanding, requiring a broad register of practical and experiential knowledge and skills. This includes the ability to “read the fish” and the sea (Lien, 2015), knowing how to respond to various indications that these readings elicit in accordance with policy or best practice (Størkersen, 2012; Thorvaldsen et al., 2018), and a thorough familiarity with the many guidelines and regulations pertaining to salmon farming and animal welfare. These guidelines include not just explicit laws on animal welfare, but a network of related and overlapping regulations regarding everything from transport to sanitation, lice counts, and working hours (Gismervik et al., 2020; Mellbye, 2018). Within this dynamic regulatory framework, salmon farmers must perform daily tasks while maintaining the welfare of the animal lives under their purview by virtue of their intrinsic value [*egenverdi*] as well as their economic value [*økonomisk verdi*]. This value dualism is by no means unproblematic (Baard, 2019; Heeger & Brom, 2001) and perhaps indicates the futility of attempting to maintain an ethically defensible IFAP aquaculture industry, free from animal suffering, within the logic of intensive capitalism (Rossi & Garner, 2014).

### Welfare courses

As one measure to ensure that fish welfare is maintained across the aquaculture industry, Norwegian animal welfare legislation requires that all fish farm workers tending the fish attend fish welfare courses similar to those in other IFAP industries in the country (Gismervik et al., 2020; Lien, 2015) at least every five years. Although held by several different organizers, welfare courses share a common regulatory framework under the national Norwegian Food Safety Authority (*Mattilsynet*), which also prescribes common guidelines for required content and learning outcomes. As far as we are aware, Norway is the only country where animal welfare courses for fish farm workers are mandated by the government. We see these welfare courses as a significant change in relation to the implementation of welfare legislation in aquaculture. Since 2010, when welfare courses became mandatory, they have provided an arena for fish farm workers to reflect on and articulate concerns about fish welfare.

Anthropological research in the Norwegian aquaculture industry has described how, although fish welfare may have been a concern among some fish farm workers, there have traditionally been few, if any, arenas in which such concerns could legitimately be raised and effectively addressed (Lien, 2015: 142–45). A similar gap between regulatory bodies and those to whom welfare responsibility is delegated has been described across agricultural industries, from pork facilities in the US (Blanchette, 2020) to cattle farms in England (Singleton, 2010). While some contend that welfare courses might constitute an intensification of governmental control, serving to relieve individual responsibility (Anneberg & Vaarst, 2018: 111), we suggest that, in their framing of welfare as a legitimate and obligatory matter of concern, the courses can also counteract such control-induced passivity, providing that they adequately draw on the experience of their participants.

Furthermore, as we detail below, these courses have become arenas in which the complex network of aquaculture regulations, especially those pertaining to animal welfare, are systematically juxtaposed and considered in relation to one another, exposing inherent contradictions.

## Research methodology

Our study was conducted in compliance with the Norwegian National Committee for Research Ethics in the Social Sciences and Humanities (NESH).^4^ Specific protocols ensuring ethical compliance were approved by the Norwegian Center for Research Data (NSD), who undertakes ethical clearance on behalf of the University of Oslo. Informed consent was obtained at two levels. First, we sought permission to attend courses and conduct the study from the institution(s) responsible for the welfare courses. Permission was granted with no obligations in relation to the institution(s). Secondly, informed consent was obtained for individual voluntary participants in our study, who were de-identified in our notes and subsequently anonymized, in accordance with NSD guidelines. Voluntary participation was achieved as follows: we identified ourselves as independent researchers to all course participants at each course before they began. Information about the study was made available to all participants in the form of an informational pamphlet that we handed out at the commencement of each course. Participants who chose to speak with us were assured that they could withdraw from the study at any time and were given appropriate information about how to do so. A similar protocol was applied for the visits to aquaculture facilities.

Welfare courses were selected as the main point of entry into research on fish farm workers’ perceptions of fish welfare in the Norwegian aquaculture industry, partly because these courses were precisely and explicitly *about* fish welfare. Moreover, due to their mandatory nature, welfare courses brought together aquaculture employees with different areas of expertise, levels of experience, professional backgrounds, and companies (albeit largely from the same geographic localities).

During 2017 and 2018, we (the first and second authors) attended five welfare courses, taking place over 2–3 days in various localities along the country’s southwestern coast, between the cities of Trondheim and Stavanger. We also conducted ten interviews with course participants, including well-boat captains, on-site aquaculture personnel, slaughterhouse employees, operations managers, and course instructors from several different companies, representing a diverse range of actors within the industry. Of the interviewees, whose ages ranged from approximately 20–50, the majority (80%) were male. All of our interlocutors were Norwegian, or had been living in Norway for several years, and all interviews were conducted in Norwegian. We did not collect individual data on educational attainment, but Norwegian fish farm workers typically have secondary education, and many also have vocational degrees relevant to maritime or aquaculture industries. Participants working in administration often hold degrees in business administration or similar.

The first author of this article also conducted two all-day field visits to aquaculture facilities to further familiarize themselves with the contexts discussed at the welfare courses, often held at locations (hotels, conference centers, schools) in close proximity to fish farms. The second author had conducted fieldwork at a fish welfare course approximately five years prior to this study (cf. Lien, 2015), and has extensive field experience from aquaculture facilities, which informed our questions and the interpretation of our data.

Our methodological approach was based on the anthropological method of *participant observation*, supplemented by semi-structured interviews and informal discussions with welfare course participants during lunches, breaks, and after courses. Our observations and data were gathered in field notes, which were subsequently parsed and analyzed. This methodological approach allowed us to be present during the entirety of the courses, learning and repeating the course material in the same way that other participants did, and participating in the same formal and informal discussions, workgroups, and activities such as quizzes. Participant observation also conferred us the benefit of being considered “one of the participants”, providing us access to discussions and conversations outside of the courses themselves, whilst maintaining a clarity about our role as independent researchers at these courses—in other words, what James Spradley (1980) terms “moderate participation.”

Informed participant observation enabled us to recruit interlocutors without much difficulty. Some course participants effectively recruited themselves, while we chose to approach others based on comments made during courses, and arrived at others through “snowball sampling”, where existing interlocutors help to recruit acquaintances. All of the interlocutors we spoke to were informed about the nature of our research, our adherence to national research ethics guidelines, and the fact that all participation was voluntary.

Welfare courses comprised advantageous field sites for our research. Based on common, government-mandated guidelines, they provided insight into how animal welfare in aquaculture was addressed by legislative bodies through regulations. For course instructors, who often had a veterinary background, they provided the opportunity to discuss scientific knowledge and governmental regulations about fish and welfare with a broad group of fish farm workers with hands-on experience with the practical implementation of regulations. For participants, courses provided information, competency, and values, and served as classrooms for a universal crash-course (or refresher) on animal welfare, but also as arenas for professional and social interactions between participants who encounter animal welfare in different parts of the aquaculture operation. Welfare courses were therefore valuable sites for exploring how fish welfare is mobilized by regulatory bodies and course instructors, but also how fish welfare concerns are encountered in the day-to-day labor of fish farm workers, thus bringing together theoretical and practical knowledge. In this article, we focus primarily on the perspectives of course participants,^5^ paying close attention to the ways that questions, statements, and concerns were formulated as well as to the ways in which the contexts (formal course settings, informal group discussions, and informal individual discussions) might affect the data gathered. We strove to prepare our questions with care. In conversations with fish farm workers, we attempted first to ask questions that elicited descriptive responses of concrete fish welfare-related situations, before encouraging farm workers to make more normative statements regarding fish welfare at their workplace.

One potential drawback of our methodological approach is that less caring attitudes towards fish welfare, existing perhaps under a veneer of compliance, might have been difficult to discern (due to the courses’ explicit focus on fish welfare and because of their mandatory nature). However, focusing on how welfare regulations are presented, discussed, and even challenged, still elicits insight into such issues as compassion fatigue and burnout, which would have been difficult to obtain by other means.

### Findings

The findings of this study are presented in two parts. The first part addresses Norwegian authorities’ legal norms and state-of-the-art knowledge on fish welfare, as seen through an analysis of the pedagogical content of the welfare courses we attended. Here, we suggest that the courses present participants with two models—one that describes ‘how things are’ (concerning fish, welfare, aquaculture, regulations), and another that describes ‘how things *ought* to be’. While the first model was primarily articulated as part of the course curriculum, the second model was developed through sharing experiences of best practice, formally and informally. In this way, welfare courses also constituted arenas where attitudes towards fish welfare were shaped, both ontologically and normatively, in terms of ‘how fish are’ and in terms of how aquaculture operations *ought* to be.

The second part of the findings concerns farm workers’ own experiences of animal welfare regulations in their everyday aquaculture operations, highlighting dilemmas as they are experienced by the workers themselves and recounted to us either during or between welfare courses. These are important, because we maintain that fish farm workers are particularly well-positioned to notice welfare challenges, as they experience contradictory demands, objectives, and regulations related to welfare in their work. They are also a group whose insight is seldom reflected in studies and guidelines, which tend to be developed and discussed mainly by other professions such as veterinarians and biologists. Meeting fish farm workers in the context of a welfare course is therefore an excellent opportunity to notice dilemmas that might not register on the radar screens of other professionals.

### Findings: Welfare courses as constitutive

While the welfare courses we attended differed in some respects (e.g. location, duration, number of participants, gender balance), all shared a common core and structure. Every course we attended began with a statement of purpose, which was to ensure that all participants were ‘on the same page’ regarding the knowledge required to secure fish welfare for salmon, and to provide the attitudes, knowledge, and skills deemed necessary to maintain good fish welfare in aquaculture operations.

### Grounding ethics, normalizing fish sentience

Following this statement of purpose, courses moved on to a discussion of ethics, drawing briefly on Pythagoras and Descartes to illustrate some of the ways that animals and animal welfare have been treated in Western philosophical discourse. At every course we attended, the course instructors made it clear that little doubt remains over the assertion that fish *are* sentient beings, capable of feeling pain. At one course, the instructor introduced this topic by asking for a show of hands from participants who believed that fish were incapable of feeling pain. No hands went up, although social pressures and participants’ knowledge of expected responses could well have influenced this unanimous response. On the whole, the question of whether fish are capable of feeling pain appeared to be settled, and less relevant than the more immediate question of “how can I avoid hurting the fish unnecessarily?” For this part, course instructors presented a review of practical procedures such as feeding, handling, stunning, counting, and killing, emphasizing not only the ways that things are normally done, but *why* they are done this way; in other words, how welfare considerations have contributed to shaping quotidian aquaculture practices.

While three of the courses moved quickly from introductions such as those mentioned above, to more recent or pragmatic welfare entanglements, two of the courses included anecdotes from the past, or from other parts of the world. These anecdotes appeared to have been included to highlight how a) perceptions of animal welfare in Norway have changed over time, and/or b) how these differ in various contexts or cultures. While the overall narrative on changing perceptions of animal welfare was one of progress (towards increased awareness of and compliance with animal welfare considerations), the cross-cultural comparisons tended to portray Norway as being at the forefront, with other countries or cultures lagging behind.

Anecdotes such as these included images and video clips of farm animals being subjected to blatant animal abuse and cruelty, eliciting appalled responses from participants. One video clip showed a group of men in stereotypically Arab garb throwing live sheep into the back of a truck, hitting, kicking, and berating those sheep who did not immediately comply. Another showed a live fish being prepared and eaten in an Asian restaurant. The course instructor commented that many of the images depicted seemed to be from Asia and the Middle East and asked rhetorically whether this meant that they were “bad people”. He asked, “Is there is big difference between these images and the way we do things in Norway?”, prompting a mix of responses from participants. The course instructor continued: “It is better here—but why?” After a short interlude, he answered his own question: “Because the Food Safety Authority is breathing down our necks—but also because we have attitudes and knowledge that these [people in the videos] haven not received. There is a need for information and education, because you must know what a fish needs—and that is something we have in Norway.”

### Ethics as cultural and legislative process

Through teaching material such as these videos, participants were invited to distance themselves from certain attitudes towards animal welfare, and concomitantly to self-identify as part of a group or culture that treated animals in a more ethically sound fashion. If Norway is imagined as better than other countries, it is made clear that this is not a result of *culture*, but of national differences in legislation and state control. Furthermore, such anecdotes suggest that although animal welfare is perceived differently in other places,’we’ are at the forefront. Just as stories about animal welfare in the past mobilize progress to explain differences over time in people’s perceptions of animal sentience, cross-cultural comparisons give the impression that some countries, or parts of the world are ‘ahead’. Animal welfare is in this way inscribed into time, location, and moral universe according to which cultures are organized hierarchically (for a similar process in relation to the story of animal domestication as a story of progress, see Lien et al., 2018). Participation in welfare courses entails participation in the work of such inscription and offers a guide to appropriate (morally sound) attitudes and behaviors, and contributes towards underpinning a sense or moral obligation amongst the participants.

As part of a historical overview of farm welfare legislation, all the courses mentioned the Brambell Report of 1965. This is a historically important document for animal welfare in IFAP contexts, as are the closely linked “Five Freedoms”: freedom from hunger or thirst, freedom from discomfort, freedom from pain, injury, or disease, freedom to express normal behavior, and freedom from fear and distress (FAWC, 1979).^6^ Course instructors often held these freedoms up as ideals (how things ought to be), before asking participants if these freedoms were present for the salmon with whom they worked (how things are).^7^ Responses to this question varied, with some course participants raising the uncomfortable question of whether *any* of these freedoms are achievable in industrial aquaculture.

All of the welfare courses reviewed national regulatory demands for animal welfare as well as those of various “welfare certification schemes” such as the RSPCA and Global Gap. There were also questions about who defines good fish welfare: veterinarians, fish biologists, consumers, journalists, the EU, “the market”? Various indicators of fish welfare (and non-welfare) as well as systems for assessing welfare on fish farms, such as the Salmon Welfare Index Model (SWIM) (Stien et al., 2012) were also reviewed and discussed in relation to data on salmon (and cleaner fish) physiology.

In each course, course instructors emphasized that fish farm workers were responsible for ensuring good welfare, and for communicating their experiences of regulations and practices that affect welfare “upward” through the hierarchical tiers of the company, even to the authorities and to legislative bodies. In this way, fish farm workers’ responsibility for fish welfare (and for situations in which welfare is compromised) was emphasized, as were their roles. These roles can involve conflicting demands, but they also lay down the legal basis of empowerment when it comes to “speaking up” and possibly also resisting the pressure to implement practices that are seen as problematic.

### “Speaking up”: participants’ agreements and concerns

Active participation in the courses varied greatly. On some occasions, some 15–20 participants remained silent, dutifully taking notes or studying the educational materials they were provided. During one course, hardly anyone volunteered responses to the instructor’s questions. At others, participants actively engaged in discussions with the course instructors and one another, and hands were frequently raised to compare best practices in the workplace and to raise concerns about fish welfare in their daily work. There was at times an underlying tension in these conversations, where participants seemed to want to relate specifics of their workplace, but perhaps felt apprehensive about doing so in front of their colleagues and, in some cases, their superiors.

Courses typically included participants from different areas of production from several different companies. Not surprisingly, we found that informal discussions in smaller groups—during lunch breaks, for instance—yielded more specific information on matters of concern, and issues that had been raised in the plenary sessions were articulated more sharply, with more affective engagement, in these more informal conversations.

Overall, the interviews and discussions during welfare courses suggested that there was a great deal of agreement about many of the common issues raised regarding fish welfare for farmed fish. Participants expressed this of their own accord or when they were prompted by course instructors to reflect on their own experiences related to animal welfare. For the most part, it seemed as though most fish farm workers agreed that the welfare of their fish was *generally* good—although, as one interlocutor said, “it could always be better”. However, there were also several instances of informal conversations when fish farmers spoke more frankly, voicing concerns about the welfare-related challenges that they experienced in their work, and sharing their frustration with a smaller group of colleagues (and the authors). In the following section, we look at these concerns in more detail and highlight some of the dilemmas that are articulated by fish farm workers.

### Findings: Fish farm worker perspectives

Welfare challenges as perceived by participants were mostly related to the way that fish welfare was safeguarded—or not—in everyday practice. The most common concerns were the welfare implications of sea lice treatments on salmon (brushing, warm water treatments, water flushing, pumping), the welfare of cleaner fish, issues related to overcrowding, long-term stressing of the salmon, and the intensification of industrial measures to increase growth, pushed by both the government and by private aquaculture actors. These were frequently related to us in solemn and sometimes emotional accounts, and we were struck by the profound sense of occupational and personal or moral responsibility for fish welfare that mobilized these accounts.

Their accounts and the way that they were related indicated that some fish farm workers saw themselves as stuck between “the devil and the deep blue sea” with regard to fish welfare, unable to balance contradictory demands. This was particularly brought to the fore by fish farm workers who were directly responsible for fish welfare on a day-to-day basis at aquaculture facilities.

### Economies of scale versus moral considerations

The contradictory demands that fish farm workers outlined can be described as a classic conflict between economic and moral considerations. On the one hand, economic demands and expectations of increased, intensified, and more efficient production instilled a sense of responsibility in fish farm workers towards contributing to these objectives. This sense of economic responsibility is supported by major economic rewards, strong competition between private aquaculture actors, and the Norwegian authorities’ stated aim of a significant increase in aquaculture production made public in a white paper in 2015 (Ministry of Trade, Industry & Fisheries, 2015; see also Lien & Law, 2019).

On the other hand, there is the personal and professional responsibility that fish farm workers harbor for the welfare of the fish they care for. It should come as no surprise that many fish farm workers acknowledge fish sentience and express empathy and affective relations for the fish they care for. This has been observed prior to the implementation of new fish welfare regulations, including mandatory welfare courses (Lien, 2015).^8^

We suggest that the introduction in 2010 of welfare legislation specific to aquaculture acted to confirm concerns about fish welfare relative to other IFAP industries in Norway. While the welfare courses themselves may not sufficiently address the underlying reasons for some fish farm workers’ concerns, they act to assert the legitimacy of such concerns and simultaneously provide an arena for their more forceful articulation and discussion. Hence, we can hear remarks such as the following, recounted by a farm worker during a welfare course:

A bit ‘too quick for its own good’ [*litt fort i svingene*] is an apt characterization of several things going on in the industry … there’s too much money in it … it’s the growth, you know, the fucking demand for continued growth. When you have business leaders that made billions of crowns, it is clear that some corners will be cut. They have to be.

Furthermore, and somewhat paradoxically, complying with a range of different and sometimes contradictory welfare regulations can create unintended and sometimes problematic consequences, even for practices ostensibly set in place to safeguard fish welfare, which is another cause of concern for several of the fish farm workers with whom we spoke.

**Fig. 1:**
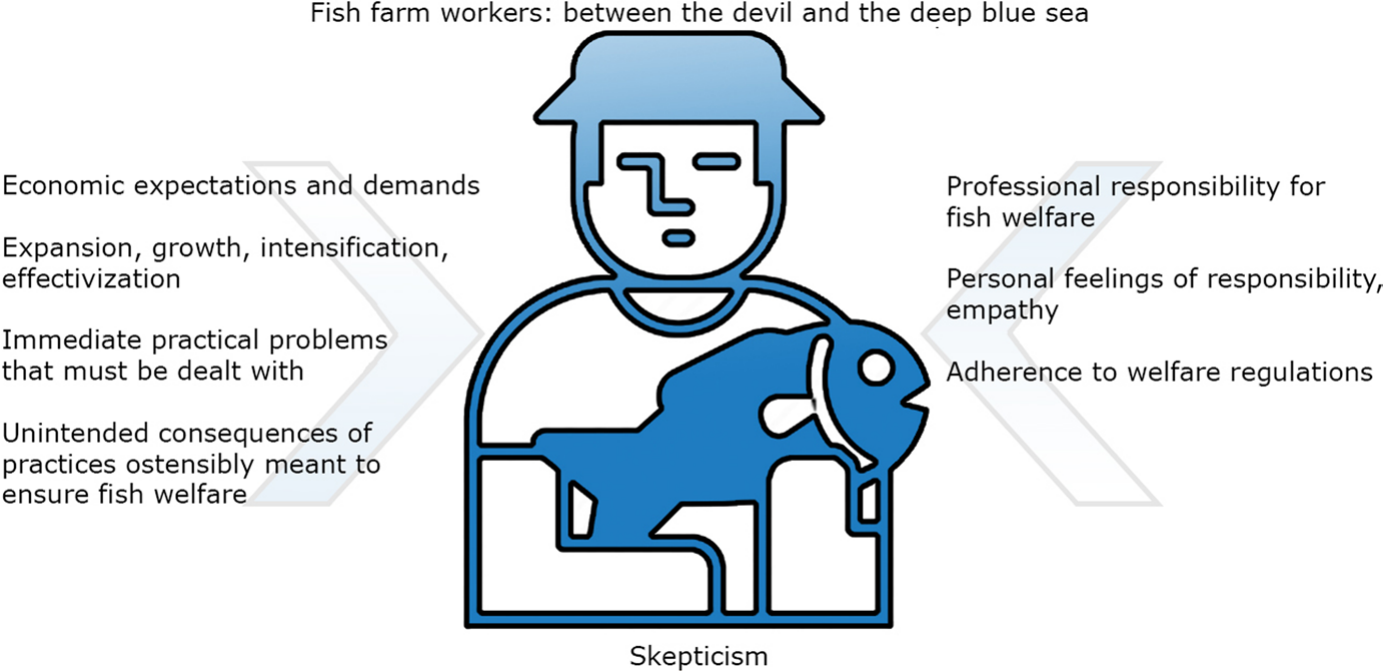
Contradictory, sometimes irreconcilable considerations

This dilemma of contradictory, sometimes irreconcilable considerations is illustrated in Fig. 1.

### (Not) Caring for cleaner fish

We suggest that what fish farm workers experience as irreconcilable considerations are best understood as indications of a structural dilemma, reflecting contradictions in the way that aquaculture is organized (Størkersen et al., 2021). The intensification of production, for example, may well be expressed as increased numbers of fish in the fish farms as well as the density of aquaculture sea cages in Norwegian fjords—both which provide ideal conditions for sea lice infestations (Taranger et al., 2015). Consequently, they require more measures to combat sea lice, such as the use of cleaner fish or other delousing treatments. According to fish farm workers, these are the instances in which fish welfare was most often compromised. As one interlocutor recounted about the use of cleaner fish:

We’ve gradually gotten to the point of having three different [types of fish] in the cages, with three different physiologies, [and] swim bladders: wrasse [closed swim bladder], lumpfish [no swim bladder], and salmon [open swim bladder]. That means a lot more work for us, because we should of course treat each type of fish differently. At the same time, we bring wrasse up [to the surface] when we bring up salmon, and they turn themselves inside out [*de vrenger seg*]—sure, it’s uncomfortable. It’s horrible.

In their final sentence, the interlocutor is referring to barotrauma, and specifically to the way that fish with closed swim bladders (or none at all) have no means of quickly regulating pressure changes brought about by rapid changes in depth. When they are suddenly brought up to the surface (often along with salmon), the air in their swim bladders expands rapidly, often causing serious internal damage to the fish and sometimes forcing their innards to emerge from their mouth and other orifices.

### Handling the “losers”

Another issue that farm workers brought up was what is referred to in the industry as “loser” fish—those which grow too slowly during their first months in the cages. These fish are systemically sorted away when they are spotted and either culled or relocated to a separate cage. Here, they wait to be “asphyxiated and ground up with the rest of the dead fish” [*daufisk*]. In this way they are made invisible through the ordering of the assembly line (Lien, 2015: 135). This suggests both a conceptual and physical separation from “healthy salmon” (those that have thrived and grown quickly in the first weeks in the cage). When asked what was upsetting about this, one interlocutor replied that sorting out the fish that do not fulfill the industry’s expectations for rapid growth was “very uncomfortable”, because they were basically sending them to an early death. Here we see how the requirements for standardization and efficiency in the highly industrialized production systems is at odds with what could be seen as natural variation of salmon in the cage. But while the “early death” was perceived as uncomfortable by one farm worker, it could also be understood as a way of preventing or alleviating pain in the future. This is in keeping with the way that the entire set-up is geared towards uniformity, a condition that inevitably marginalizes young fish who have not shown adequate rates of growth. At stake, then, is how morality changes with the change of perspective, from caring for fish as individuals (as in the comment above), to caring for a cohort, such as a cage (or tank). The latter is the perspective that guides most operations and indicates that a hierarchy is at work in which individuals less likely to survive or to grow to an economically profitable size are systematically downgraded and eliminated, in order to safeguard the uniformity of each cohort.

## Discussion

We have shown how fish farm workers find themselves in a situation of contradictory considerations and demands. The fish welfare courses bring these dilemmas out in the open, but also contribute to sharpening the affective impact of these moral tribulations. This is due, in part to an internalization of values about fish welfare and a growing consensus that fish are sentient beings, capable of feeling pain—but also to the way the welfare courses place the responsibility for fish welfare directly on farm workers. This message is transmitted both as practical advice (how to do things better), but also implicitly, through cross-cultural comparisons that make the safeguarding of animal welfare a moral imperative for “civilized” countries. We have limited historical data to compare with, but conversations with “old timers” in the industry indicate that these dilemmas are felt more strongly now than they used to be. Indeed, as one interlocutor told us, “It is not long ago that fish were seen as having less intrinsic value than terrestrial animals, and to some extent such attitudes still exist.”

Additionally, the increased push towards efficiency and profit in aquaculture operations, backed by government policy, exacerbates the intensity of this dilemma. Added to this are increased reporting requirements: counting, testing, sampling, and measuring several parameters for a range of different purposes, both welfare-related and economic. Together, these operations take up time and involve more frequent handling of the fish, which in itself may induce stress and compromise welfare on an individual level. Fish welfare in aquaculture is shaped at the interface of a range of different practices, some of which have unintended negative welfare implications. These practices are in turn responses to different aims and concerns, and different sets of legal regulations. A thorough understanding of the dilemmas that fish farm workers experience calls for an understanding of the internal contradictions and idiosyncrasies of this fish welfare assemblage—its inherent gaps—and how it unfolds in specific locations and legal contexts.

The introduction of mandatory fish welfare courses in Norway has made such idiosyncrasies more visible, as their pedagogical framing has made workers’ responsibilities more explicit. This raises several questions. First, how do idiosyncratic practices and unintended consequences occur and how might they be avoided? Second, to what extent do fish farm workers have the ability to respond in ways that may safeguard wellbeing within and around the sea cages? Third, how could the aquaculture industry as a whole better utilize the untapped potential of fish farm workers in improving fish welfare in aquaculture operations? We will consider each of these questions below.

### Idiosyncratic practices, unintended implications

Welfare courses brought to the fore several challenges experienced by fish farm workers in their daily work. Many of these challenges stemmed from organizational conditions in the industry as well as from the complex framework of regulations and objectives that apply to aquaculture operations in Norway. In contrast to other IFAP industries, such as chicken production, in which producers must comply with two laws governed by the same governmental ministry, salmon producers are pursuant to multiple regulations from two different ministries as well as several different agencies (Gismervik et al., 2020). Additionally, some of these regulations, such as those aiming to prevent and combat outbreaks of Pancreas Disease (PD), divide the country into different regional zones with different directives and practices, adding to the complexity. Fish farm workers told us that these regulations and objectives often overlap or come into conflict with one another, and as a result they experience their own position as one of multiple constraints. As we have suggested, these conflicting regulations may be described as a dilemma between production and protection (or economic and moral considerations)—but they are perhaps better understood as a multiple protection dilemma; a conflict between protecting farmed fish and protecting wild fish (Størkersen et al., 2021). This raises the question of whether fish welfare is sidelined in favor of other concerns or considerations—the welfare of the cleaner fish sacrificed for the welfare of farmed and wild salmon, the welfare of the farm worker sacrificed for the welfare of the company, the welfare of the salmon sacrificed for the welfare of shareholders?

### Responsibility and fish farm workers’ ability to respond

Fish farm workers are taught to take responsibility for the welfare of the salmon under their supervision. There is, however, a difference between responsibility and the ability to respond. Donna Haraway has proposed the term “response-ability” to denote the human condition of being both willing and capable of responding adequately to animal suffering (Haraway, 2008). Studies of care have indicated that good care involves practical relational adaptations, also described as “tinkering” (Mol et al., 2010). Such tinkering is at the heart of the everyday practices of fish farm workers and involves responding effectively and in a timely manner in ways that diminish unnecessary suffering among farmed fish. However, if an increased pressure to solve contradictory and time-consuming demands (including reporting on welfare parameters, such as lice counts and fish mortality) hampers farm workers’ ability to respond as well as they potentially could, their “response-ability” diminishes too: they may find that the strains of reporting and accountability impede their ability to provide adequate care for the fish, for instance. The effect of this is not only the emotional stress and frustration of not being able to care for salmon in accordance with their own sense of responsibility, but also an untapped potential for fish welfare improvement for the industry as a whole. This leads to the third and final point, namely the extent to which farm workers’ voices are sufficiently heard when fish farm welfare regulations are shaped and implemented.

### From industrialized indifference to institutionalized care?

In his account of hog factory farms in the United States, anthropologist Alex Blanchette gives a detailed account of the welfare implications of contemporary cost-cutting efficiency schemes in pork production. In the context of minimal welfare regulations and brutal profit maximization, the animal suffering is blatant and disturbing. However, inside this machinery he also encounters farm workers who have cultivated an affective care for pigs and piglets that contradicts the fundamental principles of the industry as a whole. He concludes:

“At a moment in which definitions of what constitutes animal welfare and the ethical treatment of farm animals tends to be decided by scientists at universities, what seems clear, at a minimum, is that workers—those who know the conditions of modern hogs most intimately—should have an intellectual say in what constitutes ‘humane’ agriculture in the first place.” (Blanchette, 2020: 152).

A similar case could be made for aquaculture. First, this implies that fish farm workers should be recognized by welfare regulators not only as key personnel for the *implementation* of welfare legislation, but also as sources of hands-on, practical, and intimate knowledge of potentially harmful situations and how they might be avoided. This involves approaching fish farm workers as intellectual contributors in their own right, whose knowledge can complement (and challenge) that of fish scientists and veterinarians as they improve regulatory protocols.

Second, it implies that fish farm workers are recognized as welfare advocates at their own workplace. More precisely, appropriate institutional channels of information and decision-making procedures must be in place that not only take the workers’ perspective “on board”, but actively elicit workers’ opinions so that fish farm workers have an internal voice. This would imply that legitimate arenas for discussing fish welfare and practical solutions are extended and multiplied, within and across the industrial sector.

Recognizing the intellectual and practical contribution of fish farm workers would also slightly reframe the mandatory welfare courses. From being primarily a pedagogical “one-way street”, they could become an arena for discussion across various industrial sites as well as an arena for up-to-date feedback *to* regulators and legislators regarding the challenges involved in implementing fish welfare regulations on a day-to-day basis, and novel challenges that unfold—for instance, in relation to delousing procedures and the plight of cleaner fish.

## Conclusion

We have pointed out how mandatory fish welfare courses represent a significant step towards fish welfare, but that they also create a kind of gap, exposing the difference between the ideals inherent in fish welfare regulations and the shortcomings of everyday practices for fish welfare in the Norwegian aquaculture industry. As these gaps are exposed, while fish farm workers are simultaneously heralded as the upholders of fish welfare, we see the contours of another dilemma, namely that of fish farm workers failing to meet the requirements that their work entails. There is a risk that such experiences of failure and shortcoming can be internalized as stress or even pain in the individual bodies of the fish farm workers, which could even be transmitted and felt by the fish as well. This article contributes to “minding” this gap. We have suggested that recognizing the untapped potential of fish farm workers in relation to the welfare of farmed fish could not only lead to significantly improved fish welfare, but also to improved working conditions for fish farm workers. Being recognized as fish experts in their own right, and given the ability to respond to feelings of (ethical) responsibility, fish farm workers might not only identify dilemmas before they cause harm, but also feel empowered and able to carry out their daily work with the “response-ability” that contemporary welfare legislation presumes.

## Data Availability

This is ethnographic data material based on interviews and participant observations. In accordance with GDPR and ethical guidelines, research participants have agreed to the use of their information through informed consent. Participants are not identifiable in the text, and their anonymity is secure. In accordance with their informed consent, while the date material is technically available, it is not legally available the purposes other than the current project.

## Footnotes

1 One important caveat to these courses is that they have not been mandatory for all high-level leaders who make decisions that affect fish welfare (Gismervik et al., 2020).

2 This in addition to the multitude of non-humans (particularly “cleaner fish”) recruited to carry out various tasks.

3 For recent critical accounts of practices related to cleaner fish in Norwegian aquaculture operations, see Lien (2017); Mo & Poppe (2019).

4 The NESH guidelines can be found here: https://www.forskningsetikk.no/en/guidelines/social-sciences-humanities-law-and-theology/guidelines-for-research-ethics-in-the-social-sciences-humanities-law-and-theology/.

5 But see Størkersen et al. (2021) for a study of organizational conditions for maintaining fish welfare in Norwegian aquaculture that draws on interviews and survey data from employees outside of welfare courses.

6 See also the Norwegian Food Safety Authority’s own page on animal welfare and the Five Freedoms: https://www.mattilsynet.no/dyr_og_dyrehold/dyrevelferd/rad_om_dyrevelferd/hva_er_dyrevelferd.5017.

7 We must add that in spite of a common misconception, repeated at the welfare courses, the Five Freedoms were not defined in the Brambell Report, but by the UK Farm Animal Welfare Committee (FAWC) in 1979,

8 See also Blanchette (2020): 153–57 for similar relations to piglets in a highly industrialized setting.
